# Essential Oil Composition and DNA Barcode and Identification of *Aniba* species (Lauraceae) Growing in the Amazon Region

**DOI:** 10.3390/molecules26071914

**Published:** 2021-03-29

**Authors:** Júlia Karla A. M. Xavier, Leonardo Maia, Pablo Luis B. Figueiredo, Adriana Folador, Alessandra R. Ramos, Eloísa H. Andrade, José Guilherme S. Maia, William N. Setzer, Joyce Kelly R. da Silva

**Affiliations:** 1 Programa de Pós-Graduação em Química, Instituto de Ciências Exatas e Naturais, Universidade Federal do Pará, Belém, PA 66075-900, Brazil; julia.xavier@icen.ufpa.br (J.K.A.M.X.); leo_henriquemaia@hotmail.com (L.M.); 2 Departamento de Ciências Naturais, Centro de Ciências Sociais e Educação, Universidade do Estado do Pará, Belém, PA 66050-540, Brazil; pablo.figueiredo@uepa.br; 3 Laboratório de Genômica e Bioinformática, Centro De Genômica e Biologia de Sistemas, Universidade Federal do Pará, Belém, PA 66075-900, Brazil; adrianarc@ufpa.br; 4 Instituto de Estudos em Saúde e Biológicas, Universidade Federal do Sul e Sudeste do Pará, Marabá, PA 68507-590, Brazil; rezende@unifesspa.edu.br; 5 Coordenação de Botânica, Museu Paraense Emílio Goeldi, Belém, PA 66040-170, Brazil; eloisa@museu-goeldi.br; 6 Programa de Pós-Graduação em Química, Universidade Federal do Maranhão, São Luís, MA 64080-040, Brazil; gmaia@ufpa.br; 7 Department of Chemistry, University of Alabama in Huntsville, Huntsville, AL 35899, USA; wsetzer@chemistry.uah.edu; 8 Aromatic Plant Research Center, 230 N 1200 E, Suite 102, Lehi, UT 84043, USA

**Keywords:** *Aniba* species, terpenoids, benzenoids, DNA barcode, genetic variability

## Abstract

Lauraceae species are widely represented in the Amazon, presenting a significant essential oil yield, large chemical variability, various biological applications, and high economic potential. Its taxonomic classification is difficult due to the accentuated morphological uniformity, even among taxa from a different genus. For this reason, the present work aimed to find chemical and molecular markers to discriminate *Aniba* species collected in the Pará State (Brazil). The chemical composition of the essential oils from *Aniba canelilla*, *A. parviflora*, *A. rosaeodora*, and *A. terminalis* were grouped by multivariate statistical analysis. The major compounds were rich in benzenoids and terpenoids such as 1-nitro-2-phenylethane (88.34–70.85%), linalool (15.2–75.3%), α-phellandrene (36.0–51.8%), and β-phellandrene (11.6–25.6%). DNA barcodes were developed using the internal transcribed spacer (ITS) nuclear region, and the *matK*, *psbA-trnH*, *rbcL*, and *ycf*1 plastid regions. The markers *psbA-trnH* and ITS showed the best discrimination for the species, and the phylogenic analysis in the three- (*rbcL *+ *matK* + *trnH − psbA* and *rbcL* + *matK* + *ITS*) and four-locus (*rbcL* + *matK* + *trnH − psbA* + ITS) combination formed clades with groups strongly supported by the Bayesian inference (BI) (PP:1.00) and maximum likelihood (ML) (BS ≥ 97%). Therefore, based on statistical multivariate and phylogenetic analysis, the results showed a significant correlation between volatile chemical classes and genetic characteristics of *Aniba* species.

## 1. Introduction

Lauraceae Juss comprises the most diverse family of woody plants (except the herbaceous parasite *Cassytha*), with about 50 genera and approximately 2500 to 3000 species distributed throughout tropical and subtropical latitudes [1,2,3]. Lauraceae belongs to the Laurales order and systematically forms close relationships with Hernandiaceae and Monimiaceae [1,4,5].

The *Aniba* genus is a Lauraceae member that presents an economical and significant ecological value [6]. This genus has 48 accepted species with greater than 50% concentrated in the Brazilian Amazon [7,8]. *Aniba* species are represented by trees with high essential oil production in all tissues, mainly in the wood and bark. *Aniba duckei* Kosterm and *A. rosaeodora* Ducke are known as “rosewood” in the Amazon region, and these species produce an oil rich in linalool (about 85–90%) [9,10]. Brazilian rosewood oil has a characteristic aroma and is a long-established ingredient of fragrances, flavors, and food products [11]. The species *Aniba parviflora* (Meisn.) Mez, called “macacaporanga” or “louro rosa,” is confused with the real rosewood plants due to their morphological similarity. However, these species present very distinct aromas in wood and leaf oils because the linalool content in *A. parviflora* is only 40% [9,12,13]. Linalool is also detected in oils from *Aniba terminalis* Ducke, with amounts varying from 22.1 to 36.2% in the aerial parts and inflorescences of a specimen collected in Belém (PA, Brazil) [14].

*Aniba canelilla* (H.B.K.) Mez is an aromatic plant with a characteristic odor, which is easily confused with cinnamon trees. It is popularly known as “casca-preciosa” and “falsa-canela.” Its chemical composition has two main constituents, 1-nitro-2-phenylethane, which is generally found as the major component responsible for the cinnamon-like odor characteristic, and methyleugenol [15,16,17]. The *A. canelilla* essential oil EO displayed cardiovascular activities in normotensive rats, causing hypotension, bradycardia, and vasorelaxant effects [18,19,20,21,22]. The EO of *A. rosaeodora* and *A. parviflora* species showed antidepressant activity in rats [23], and an anesthetic potential in fish and rat species [24,25]. In folk medicine, the leaves and barks of *A. parviflora* are used to prepare tea and infusions, tinctures, and poultices to treat snakebite envenomation victims [26]. Additionally, the *A. canelilla* bark decoction is commonly used for its antispasmodic, digestive, stimulating, and carminative properties [16].

The recognition of the Lauraceae taxa, and the *Aniba* genus, in particular, is a difficult task due to the lack of morphological characters that can be used objectively and also due to the low level of sampling in the Amazon, which makes most species poorly represented in herbaria [27,28,29,30]. Some factors also hamper accurate identification: The use of common names for the species, which sometimes do not correspond to the scientific name; the rare collecting of fertile specimens, either for comparison in herbaria or by experts [31]. The identification methods have recently been gradually expanding to new techniques such as DNA-based identification [2,32].

DNA barcoding is designed to provide a fast, accurate, and automated identification of species using short and standardized genes as internal species markers [33]. Various molecular markers have been analyzed to develop plant DNA barcodes that can be readily sequenced and have a sufficiently high sequence divergence at the species-level [34,35]. These markers include the coding plastid regions, *matK*, *rbcL*, non-coding *trnH-psbA* intergenic spacer, and nuclear ribosomal internal transcribed spacer region (ITS) [36,37,38]. The DNA barcoding has been an essential tool for species identification and supplements traditional morphology-based taxonomy [33,39,40].

The identification of *Aniba* species by molecular markers is still unresolved. The present study aimed to explore the correlation between the volatile compositions and the genetic markers in *Aniba* species existing in the Amazon.

## 2. Results and Discussion

### 2.1. Chemical Composition and Multivariate Analysis

The yields and volatile compositions of the *Aniba* oils are displayed in Table 1. The oil yields of these species were as follows: *Aniba canelilla*, *A. parviflora*, *A. rosaeodora*, and *A. terminalis*. GC and GC-MS were used to quantify and identify the volatile constituents of *Aniba* oil samples. One hundred and eighteen components were identified, representing an average of 97.8% of the total percentage identified among the samples (see Table 1). *A. parviflora* oils showed the highest number of compounds identified, fifty-four and fifty-seven in the leaves (AP-L) and twigs (AP-T), respectively. The sample with the lowest number of compounds was *A. canelilla,* with fifteen compounds in the leaves (AC-L) and twenty-five in the twigs (AC-T). The species *A. terminalis* showed forty-two (AT-L) and forty-four (AT-T), while *A. rosaeodora* presented thirty-seven (AR-L) and thirty-eight (AR-T). The calculated retention index (RI_C_) of components of the oils were compared with the literature retention index (RI_L_) stored in the libraries of Mondello [41] and Adams [42]. Benzenoid compounds (0.1–91.8%), monoterpene hydrocarbons (0.0–88.9%), and oxygenated monoterpenes (1.2–81.7%) predominated in oils, followed by oxygenated sesquiterpenes (0.9–19.2%) and sesquiterpene hydrocarbons (0.6–10.9%), with minor amounts. The main constituents were 1-nitro-2-phenylethane and linalool in *A. canelilla;* β-phellandrene, and α- and β-pinene in *A. parviflora*; linalool and *cis*-linalool oxide in *A. rosaeodora*; and α-phellandrene, *p*-cymene, and β-phellandrene in *A. terminalis*. 

The leaf and twig oils (AC-L and AC-T, respectively) of *Aniba canelilla* were dominated by the benzenoid 1-nitro-2-phenylethane (88.3% and 70.90%, respectively), followed by the oxygenated monoterpene linalool, which showed higher amounts in the twigs (16.1%) in comparison to the leaves (3.9%). In the leaf and twig oils (AP-L and AP-T, respectively) of *Aniba parviflora*, the monoterpene hydrocarbon β-phellandrene (22.6% and 25.4%, respectively) and the oxygenated monoterpene linalool were the primary constituents, followed by α-pinene (10.6% and 4.7%), β-pinene (6.4% and 3.5%), and myrcene (3.2% and 4.1%), respectively. Significant amounts of linalool were detected in the leaves and twigs (AR-L, 67.9%; AR-T, 75.3%, respectively) of *Aniba rosaeodora*, followed by its oxygenation products, *cis*-linalool oxide (leaves, 5.4%; twigs 2.6%) and *trans*-linalool oxide (leaves, 4.9%; twigs 2.5%). The oils of leaves and twigs (AT-L and AT-T, respectively) of *Aniba terminalis* showed the monoterpene hydrocarbons α-phellandrene (51.8% and 36.0%), *p*-cymene (12.0%, 7.5%), and β-phellandrene (11.6%, 11.9%), as the primary components, respectively, followed by α-pinene (4.3%, 3.8%), β-pinene (1.5%, 3.6%), respectively, and myrcene (3.6%) only in the twigs (AT-T). Furthermore, in the twigs of *A. terminalis*, linalool (19.0%) was also identified, which was absent in its leaves. 

**Figure 1:**
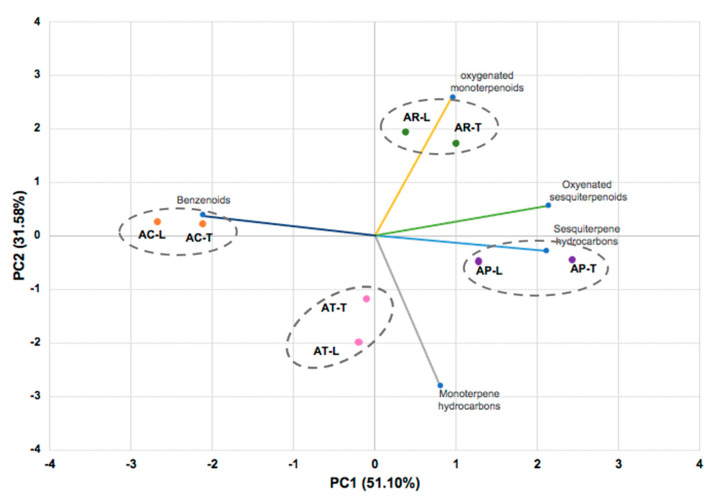
The bidimensional plot of the first two components from principal component analysis (PCA) of *Aniba* species, based on the classes of compounds present in their essential oils: **AC** (*A. canelilla*), **AP** (*A. parviflora*), **AR** (*A. rosaeodora*), **AT** (*A. terminalis*), **L** (leaves), **T** (twigs), **PC1** (first principal component), **PC2** (second principal component).

Principal component analysis (PCA) of the *Aniba* oils showed that PC1 and PC2 components had explained 82.7% of the phytochemical variation among all samples, classified into four groups (Figure 1). The PC1 component explained 51.1% of the variation, displaying a positive correlation with all terpenoid classes and a negative correlation with the benzenoid compounds. The more informative contributions to group separation were observed with the oxygenated sesquiterpenoids (30.4%), benzenoids (29.7%), and sesquiterpenes hydrocarbons (29.6%). Due to the significant content of sesquiterpenoids (11.20–30.10%) in the samples, AP-T (2.43), AP-L (1.28), AR-T (0.38), and AR-L (1.00) were represented by positive scores in the PC1 component. On the other hand, the most negative scores characterized the oil samples of *A. canelilla* AC-L (−2.68) and AC-T (−2.12) due to the significant content of benzenoid compounds (72.3–91.8%) in oils. The PC2 component explained 31.58% of the chemical variability, and the most informative contributions to sample separation were the contents of monoterpene hydrocarbons (52.1%, negatively) and oxygenated monoterpenoids (44.4%, positively), presenting in these samples in an amount around 80%. These influences can be easily visualized in the PC2 component by the samples of *A. terminalis* (AT-L, −2.00; AT-T, −1.19) and *A. rosaeodora* (AR-L, 1.72; AR-T, 1.92), respectively.

**Figure 2:**
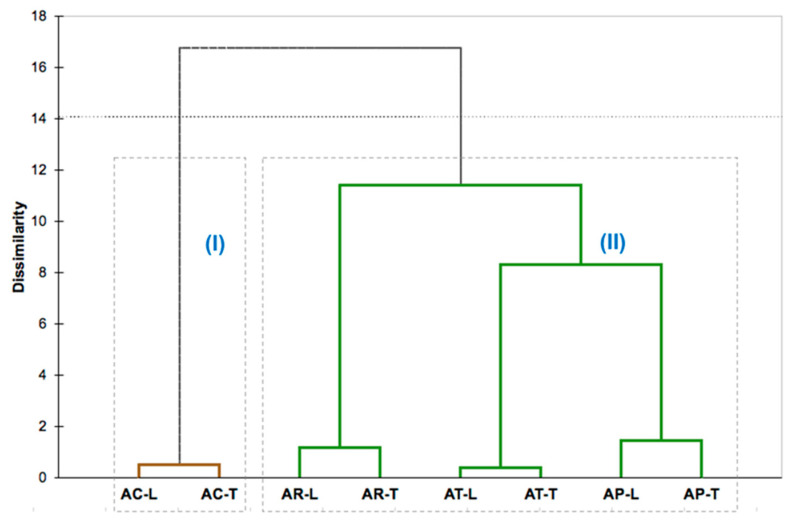
Hierarchical clusters analysis (HCA) obtained by Ward linkage method to *Aniba* species based on compound class present in their essential oil: **AC** (*A. canelilla*), **AP** (*A. parviflora*), **AR** (*A. rosaeodora*), **AT** (*A. terminalis*), **L** (leaves), **T** (twigs).

Based on the dendrogram (Figure 2) resulting from the hierarchical cluster analysis (HCA), using the classes of compounds as variables, the *Aniba* species’ oils were arranged into two main groups, presenting a dissimilarity of 16.8%. Cluster I comprised the leaves (AC-L) and twigs (AC-T) of *A. canelilla* and comprised samples rich in benzenoid compounds (72.28–91.78%), especially 1-nitro-2-phenylethane, showing a dissimilarity of 99.5%. Cluster II grouped all oil samples of *A. parviflora*, *A. terminalis*, and *A. rosaeodora*. The oils of *A. parviflora* (AP-L and AP-T) showed a dissimilarity of 8.3% with the oils of *A. terminalis* (AT-L and AT-T), forming a subgroup that displayed a dissimilarity of only 11.39% with the oils of *A. rosaeodora* (AR-L and AR-T). This second group was characterized by the high content of monoterpene hydrocarbons (47.54–88.91%) as α-phellandrene (36.0–51.83%), which is present in the oils of AT-L and AT-T; as β-phellandrene (11.58–25.58%) in the oils of AP-L, AP-T, AT-L, and AT-T; and as linalool (15.23–75.30%), the primary constituent in the oil samples of AR-L, AR-T, AP-L, and AP-T. 

Due to the morphological similarity, the species *A. parviflora* and *A. rosaeodora* have been confused concerning their true botanical identification. On the other hand, these species presented distinct scents, despite the significant linalool content in their oils. An olfactory analysis of EOs from *A. rosaeodora* and *A. parviflora*, performed by enantioselective gas chromatography coupled to olfactometry, showed a significant difference between these oils [12,43]. In addition, the aromas extracted from the leaves of *A. parviflora* and *A. rosaeodora* by solid-phase microextraction (SPME), monitored by electrospray ionization mass spectrometry, and followed by PCA multivariate statistical analysis, showed a high efficiency at distinguishing the samples, as well as at separating them by collecting data, indicating the influence of the maturation stage on their chemical composition [44]. Leaf oils from thirty-five trees of *A. rosaeodora* growing in the Pará state, Brazil, were extracted and analyzed by GC and GC-MS. Significant variations in the oil yield (1.2 to 4.2%) and linalool content (38.5–71.0%) were observed in these tree samples. On the other hand, the oil yield (0.9 to 1.3%) and linalool content (12.6 to 21.3%) determined for two trees of *A. parviflora* were much smaller, probably due to different botanical species, but with very similar morphology. Moreover, the hierarchical cluster analysis (HCA) showed differences in *A. rosaedora* and *A. parviflora* oil compositions. The oil of *A. parviflora* also showed a high content of β-phellandrene (21.1–23.6%), which is absent in the oil of *A. rosaeodora* [45].

### 2.2. DNA Barcode Analysis

According to the CBOL (Consortium for the Barcode of Life) (www.ibol.org/phase1/cbol accessed on 7 February 2021) plant working group, an ideal DNA barcode should associate conserved regions with a universal primer design, present elevated rates of PCR amplification and sequencing, and have genetic variability. This is sufficient to distinguish sequences at the species level still sufficiently conserved among individuals of the same species [36,46,47]. 

In the present study, the *rbcL* plastid DNA region exhibited a high performance, with 100% of successful reactions and specific amplifications, resulting in high-quality and straightforward sequencing for the *Aniba* species. The success of *rbcL* should be expected as it is a stable, easily amplified, and phylogenetically conserved locus [47,48,49]. Similar patterns have been reported for 133 species of Lauraceae collected in China, represented by 12 genera, including *Alseodaphne*, *Cinnamomum*, *Cryptocarya*, *Lindera*, *Litsea*, *Machilus*, and *Neolitsea*. The samples showed a *rbcL* PCR amplification and elevated sequencing rates, which were considered as barcode loci [37].

Primer universality is a critical factor unquestionably determining the reliability of barcode-based species identification [50]. The intergenic spacer *psbA-trnH* was highly successful in amplification and the intermediate reactions in sequencing. It is considered a robust marker that allows PCR amplification from diverse plant taxa [49] and special phylogenetic studies in Lauraceae [51,52,53,54]. 

The *matK* marker showed easy amplification of the samples, but the sequencing was not satisfactory. Many studies questioned its utility as a barcode due to low amplification, sequencing performance, and problems related to the primers universality [55]. Consequently, the literature has recommended a more significant number of primers from the *matK* region [49,56]. This DNA region was tested in Amazonian tree species, including Lauraceae, and the results showed a low rate of sequencing success, even after using two different pairs of primers [57]. 

Primers of two ITS regions were tested, but only the ITS-2 region demonstrated success in amplification and sequencing. Amplification difficulties of the ITS region were also found in the *Sassafras* species collected in Taiwan and 42 species from diverse genera of Lauraceae, such as *Aniba*, *Cinnamomum*, *Endlicheria*, *Laurus*, *Lindera*, *Litsea*, and *Machilus*, collected from the Chinese provinces of Hubei, Jiangxi, Guangdong, and Guangxi [58,59]. Despite multiple amplification attempts with varying DNA concentrations and annealing temperatures, the *ycf*1 marker did not amplify the tested species. The results suggested that re-development of the *ycf*1 barcode specific for *Aniba* species may be necessary.

Based on the markers used in this study, it was not possible to obtain the identification level for the species of *Aniba*. However, we must consider that only a few sequences of the *Aniba* genus are available from Genbank and BOLD, which served as a central reference for comparisons [60]. The best-match molecular identification was at the family level for *psbA-trnH*, *matK*, and ITS markers, and the genus level for *rbcL*.

Multiple alignments of all sequences for each region presented the most extensive length (833 bp) to *rbcL* and the shortest length (249 bp) to ITS (Table 2). In terms of molecular variation, *matK* and *rbcL* demonstrated more conserved sequences with the number of polymorphic sites (0 and 5, respectively) and nucleotide diversity showing low values (≥0.0034). The *rbcL* region is considered a benchmark locus in phylogenetic investigations by providing a taxon’s reliable placement into a plant family and genus. Therefore, it showed insufficient sequence variation to distinguish between closely related species [61].

The *matK* region also showed surprisingly low genetic diversity when analyzing 48 species belonging to different genera of the Lauraceae family, such as *Actinodaphne*, *Aniba*, *Laurus*, *Lindera*, *Litsea*, *Neolitsea*, and *Nectandra* [62]. However, in a multilocus approach, *rbcL* and *matK* discriminated a total of 49% of the 100 taxa belonging to flowering plants, including species from the Asteraceae, Caryophillaceae, Fabaceae, Euphorbiaceae, Brassicaceae, and Ericaceae [63].

The barcodes ITS and *psbA-trnH* showed the highest number of polymorphic sites and nucleotide diversity with values of 27/0.0543 and 26/0.0336, respectively (Table 2). Studies conducted with ITS and *psbA-trnH* show that both loci are powerful for differentiating Apocynaceae species [64]. The *psbA-trnH* region has been used as a DNA barcode to authenticate several plants with similar morphological characteristics [58,65]. This region in the chloroplast genome is described as a barcode rich in simple sequence repetitions (mainly stretches of mononucleotides) and small insertions and deletions (INDELs) [47,66] that lead to species-level identification of plant taxa [67,68]. The *psbA-trnH* INDEL polymorphisms served as markers to identify different species of the genus *Citrus* and *Sonneratia*, belonging to the families Rutaceae and Lythraceae, respectively [67,68].

The genetic variabilities of two populations of *Aniba rosaeodora* in the Reserva Extrativista Tapajós-Arapiuns (RESEX) and Floresta Nacional do Tapajós (FLONA), Brazilian Government conservation units in Western Pará State, were evaluated. The results showed that the *psbA-trnH* region had the highest number of polymorphism sites (25 variables) in comparison to the regions *psbD-trnT*, *trnC-rpoB*, and *trnS-trnG*, which presented values from zero to four sites [69].

**Figure 3:**
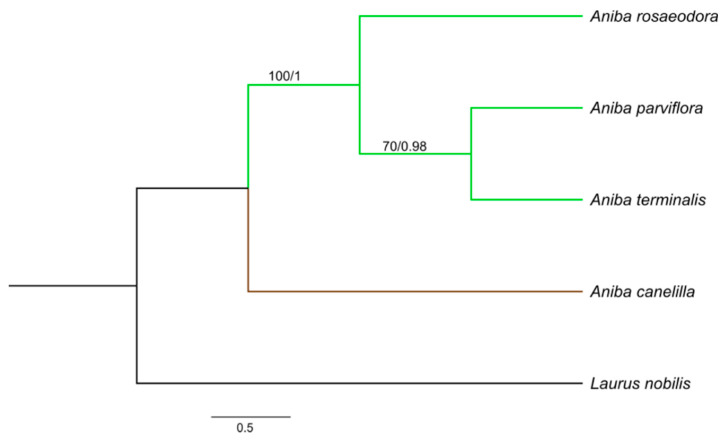
Bayesian consensus tree based on *rbcL + matK + trnH – psbA +* internal transcribed spacer (ITS) sequences of species of *Aniba* and *Laurus nobilis* (outgroup). Bootstrap support values (≥70%)/Bayesian posterior probabilities (>0.9) are shown above the branches.

**Figure 4:**
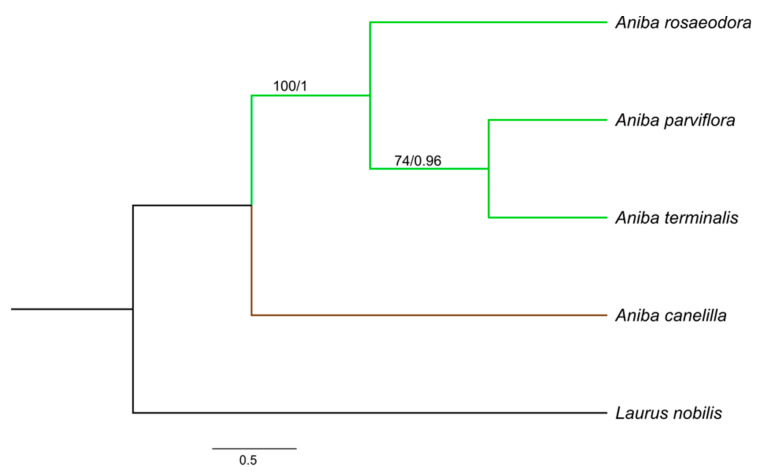
Bayesian consensus tree based on *rbcL* + *matK* + ITS sequences of species of *Aniba* and *Laurus nobilis* (outgroup). Bootstrap support values (≥74%)/Bayesian posterior probabilities (>0.9) are shown above the branches.

**Figure 5:**
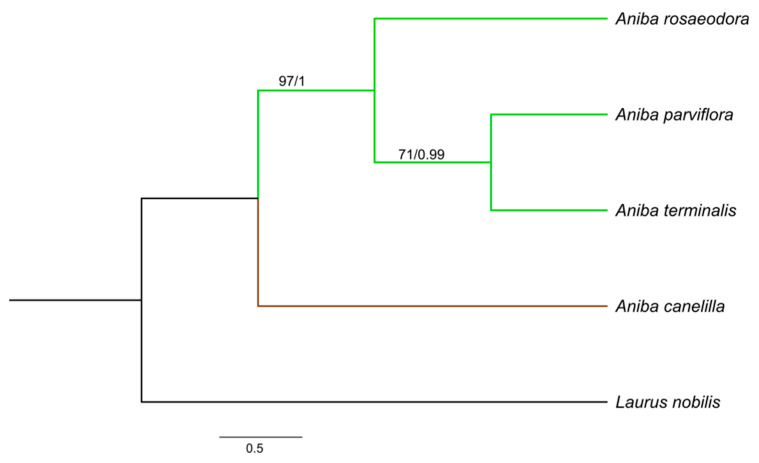
Bayesian consensus tree based on *rbcL* + *matK* + *trnH* − *psbA* sequences of species of *Aniba* and *Laurus nobilis* (outgroup). Bootstrap support values (≥70%)/Bayesian posterior probabilities (>0.9) are shown above the branches.

The aligned and concatenated matrices *rbcL* + *matK* + *psbA − trnH*, *rbcL* + *matK* + ITS, and *rbcL* + *matK* + *psbA − trnH* + ITS presented a total of 1590, 1368, and 1839 bp, respectively. Among the three concatenated matrices, *rbcL* + *matK* + *psbA − trnH* + ITS reached a greater number of polymorphic sites (58) and nucleotide diversity (0.0167). The best-fit substitution model for each gene was: HKY + F for ITS, F81 + F + I for *matK* and *rbcL*, and GTR + F for *psbA − trnH*. The phylogenetic relationships in *Aniba* species were established by the combination of *rbcL + matK* + ITS, *rbcL* + *matK* + *psbA − trnH*, and *rbcL* + *matK* + *trnH − psbA* + ITS. The consensus trees obtained from the Bayesian inference (BI) and maximum likelihood (ML) analyses were identical in their topologies, and the PP (posterior probabilities) and BS (bootstrap support) values (Figure 3, Figure 4 and Figure 5). The trees constructed by the ML and BI method for each individual region and their support values can be visualized in the Supplementary Material (Figures S1–S8).

*Aniba* species formed clades with groups strongly supported by Bayesian inference (BI) (PP:1.00) and maximum likelihood (ML) (BS ≥ 97%). *Aniba parviflora* and *A. rosaeodora* are botanically very similar species and, together with *A. terminalis*, exhibit chemical alliances due to linalool’s presence in their essential oils [11,14]. Phytochemically analyzing other secondary compounds, the presence of pseudoalkaloid anibine firmly links the species *A. rosaeodora* and *A. parviflora*, forming a complex [28,70].

In this study, *A. rosaeodora* is a sister to a clade composed of *A. terminalis* and *A. parviflora*. In addition, genetically and chemically, *A. parviflora* and *A. terminalis* have showed close relationships. The content of linalool detected in these species ranged from 15.23 to 21.93%, while that of *A. rosaeodora* was above 67%. *Aniba canelilla* presented a distinct clade as this species exhibits a high morphological similarity with *Aniba ferrea* Kubitzki. This pair of species is closely related to the exclusive presence of pyrones versus neolignans. Moreover, there are substantial quantities of eugenol-derived allylphenols [70].

The genetic and essential oil diversity was investigated with specimens of *A. rosaeodora* from Reserva Extrativista Tapajós-Arapiuns (RESEX), Floresta Nacional do Tapajós (FLONA), and the Municipality of Presidente Figueiredo, in the Amazonas State, Brazil. The analysis was performed with the concatenated matrix of the regions *psbA-trnH*, *psbD-trnT*, *trnC-rpoB*, and *trnS-trnG* forming two clades: (1) including collections from FLONA and the Municipality of Presidente Figueiredo, and (2) including collections from RESEX. Clade 1 showed moderate support (PP: 0.87), coinciding with the low diversity of volatile components from FLONA samples, where linalool predominated with 83.7%. By contrast, clade (2) was strongly supported by samples of RESEX (PP: 1.0), presenting monoterpene hydrocarbons (40.0%) and oxygenated monoterpenoids as most abundant (41.3%), with only 39.6% of linalool, followed by 22.8% of α-phellandrene, compound that was absent in the FLONA samples [69].

By contrast, this correlation was not observed in essential oil compositions and matK sequences of *Ocotea caudata* (Nees) Mez., *O. cujumary* Mart., and *O. canaliculata* (Rich.) Mez. from Caxiuanã National Forest (Amazon, Brazil). *Ocotea caudata* was characterized by the presence of germacrene D (19.9%) and monoterpene hydrocarbons α-pinene (9.8%) and β-pinene (9.7%). Simultaneously, *O. cujumary* and *O. canaliculata* showed a high similarity due to the amounts of β-caryophyllene (22.2% and 18.9%, respectively). However, genetically, *O. cujumary* was shown to be the clade’s sister (BS:70%) formed by *O. caudata* and *O. canaliculata* [71].

## 3. Materials and Methods

### 3.1. Plant Material

The specimens of *Aniba parviflora* (Nees) Mez, *A. rosaeodora* Ducke, and *A. canelilla* (Kunth) Mez were collected in the Campus of Universidade Federal Rural da Amazônia (UFRA), and the *Aniba terminalis* Ducke was sampled in the Zoobotanical Park of Museu Paraense Emilio Goeldi (MPEG), both Federal Institutions located in Belém city, Pará state, Brazil. The plant vouchers were identified and cataloged in the Herbarium João Murça Pires of Emilio Goeldi Museum, as listed in Table 3.

### 3.2. Essential Oil Extraction

The leaves and twigs were dried for two days at room temperature and then subjected to essential oil distillation. The samples were ground and submitted to hydrodistillation using a Clevenger-type apparatus (3 h). The oils were dried over anhydrous sodium sulfate, and the yields were calculated based on the dry weight of the plant material. The moisture content of each sample was measured using an infrared moisture balance for water loss measurement.

### 3.3. GC-MS Analysis 

The oil samples were analyzed on a GCMS-QP2010 Ultra system (Shimadzu Corporation, Tokyo, Japan), equipped with an auto-injector (AOC-20i). The parameters of analysis were: A silica capillary column Rxi-5ms (30 m × 0.25 mm; 0.25 μm film thickness) (Restek Corporation, Bellefonte, PA, USA); injector temperature: 250 °C; oven temperature programming: 60–240 °C (3 °C/min); helium as carrier gas, adjusted to a linear velocity of 36.5 cm/s (1.0 mL/min); splitless mode injection of 1 μL of sample (oil 5 μL:hexane 500 μL); ionization by electronic impact at 70 eV; ionization source and transfer line temperatures at 200 and 250 °C, respectively. The mass spectra were obtained by automatically scanning every 0.3 s, with mass fragments in the range of 35–400 *m*/*z*. The quantitative data regarding the volatile constituents were obtained by peak-area normalization using a GC 6890 Plus Series (Agilent, Wilmington, DE, USA), coupled to a flame ionization detector (FID), operated under similar GC-MS system conditions.

The retention index was calculated for all volatile components using a homologous series of C_8_–C_20_ *n*-alkanes (Sigma-Aldrich, St. Louis, MI, USA), according to the linear equation of Van den Dool and Kratz [72]. The components of oils were identified by comparing their retention indices and mass spectra (molecular mass and fragmentation pattern) with data stored in the NIST [73], Mondello [41], and Adams [42] libraries. 

### 3.4. Statistical Analysis

Each class of compound content in the leaf samples was used as a variable in multivariate analysis. First, the matrix’s data standardization was performed by subtracting the mean and dividing it by the standard deviation. For hierarchical cluster analysis (HCA) and principal component analysis (PCA), the Ward distance and a correlation matrix were applied, respectively. These analyses were performed using XLSTAT software (free trial version version 2021.1, Addinsoft, Paris, France). 

### 3.5. Oligonucleotides Design

The primers *rbcL* and ITS were designed using the software Primer Blast (https://www.ncbi.nlm.nih.gov/tools/primer-blast/ accessed on 20 December 2020) based on the *Aniba* sequences previously deposited on GenBank (https://www.ncbi.nlm.nih.gov/ accessed on 5 December 2020). The primers of *psbA-trnH*, *matK*, and *ycf*1 regions were based on studies described in the literature [48,72,74,75]. The primers were synthesized by the companies Síntese Biotecnologia (ITS, *psbA-trnH* and *rbcL*; Belo Horizonte, Brazil) and Gbtoligos (*ycf*1 and *matK*, Alvorada, Brazil).

### 3.6. DNA Extraction, Amplification, and Sequencing

Genomic DNA was extracted from 100 mg of fresh leaves using a plant DNA isolation Kit (PureLink™ Genomic DNA, Invitrogen, Carlsbad, CA, USA), according to its specifications, and stored at −20 °C. Polymerase chain reactions (PCR) of the regions ITS, *matK*, *psbA-trnH* e *rbcL*, and *ycf*1 were performed at a volume of 50 µL containing 4.4 µL of DNA template, 0.2 pmoles of each primer, and 43.6 µL of PCR SuperMix (22 nM Tris-HCl at pH 8.4, 55 mM KCl, 1.65 mM MgCl_2_, 220 µM dGTP, 220 µM dATP, 220 µM dTTP, 220 µM dCTP, and 22 U/mL Taq DNA polymerase, Invitrogen, Carlsbad, CA, USA). To regions ITS, *psbA-trnH*, and *ycf*1, 0.4 µL of MgCl_2_ (Invitrogen, Carlsbad, CA, USA) was added in the PCR reaction, resulting in a final concentration of MgCl_2_ of 1.84 mM. DNA amplifications were conducted in a thermocycler (GeneAmp PCR System 9700, Foster, CA, USA), and a negative control was carried out for all PCR reactions in the absence of DNA. Amplification products were visualized in agarose gel 1.5% and purified following the GeneJET PCR Purification Kit (Life Technologies, Massachusetts, CA, USA), and then sent to the ACTgene company (Alvorada, Brazil) for DNA sequencing. Table 4 presents the sequences of the primers of each fragment and its PCR amplification conditions. 

### 3.7. Sequence Identity and Phylogenetic Analysis

The forward and reverse sequences of each amplified region (ITS, *matK*, *psbA-trnH*, and *rbcL*) were edited and aligned using the software MUSCLE algorithm [76] implemented within MEGA 7 software [77]. Sequences were compared with available sequences in the National Center for Biotechnology Information (NCBI) GenBank database (http://www.ncbi.nlm.nih.gov/ accessed on 7 December 2020), using the tool Blast N. DNA sequences generated in this study were deposited in the NCBI GenBank, and accession numbers are listed in the Supporting Information (Table 5).

DNA sequences were aligned using the software MAFFT Ver 7.122 (Osaka University, Osaka, Japan) [78] and manually adjusted, when necessary, with the MEGA7 software [77]. The phylogenetic analyses were inferred from sequence variation in the three-locus (*rbcL + matK + trnH−psbA* and *rbcL + matK* + ITS) and four-locus combination *(rbcL + matK + trnH − psbA* + ITS). The analyses were performed in the software PhyloSuite (Github, Free Software Foundation, Inc., San Francisco, CA, USA) [79] using two different approaches: Maximum likelihood (ML) and Bayesian inference (BI) analyses using the programs IQ-Tree v.6.1 (Github, Free Software Foundation, Inc.) [80] and MrBayes v.3.2.6 (Github, Free Software Foundation, Inc.) [81], respectively. Evolutionary models were tested using the ModelFinder program implemented in IQ-TREE version 1.5.4 (Github, Free Software Foundation, Inc.) [82], based on the Akaike information criterion (AIC). For the ML and BI analyses, the dataset was partitioned by markers. For maximum likelihood analyses, the branch supports for the tree were estimated with 5000 bootstrap replicates using UFBoot (Ultrafast Boostrap Approximation) [80].

For Bayesian Inference, analyses were performed using two parallel runs and a sampling frequency set to every 10,000,000 generations. The trees were sampled every 100 generations, and the first 25% of the samples were discarded as burn-in trees. The remaining trees were used to construct a 50% majority-rule consensus tree. *Laurus nobilis* L. was defined as an outgroup (KM360844.1, MH552343.1, AY265392.1, and EU153959.1). The resulting trees from both analyses were output into FigTree v.1.4.4 [83], and their topologies were compared.

The median length described the genetic variability of each marker (bp) and total alignment length (bp), both discounting gaps, the number of sites with gaps, and nucleotide diversity (π), using the DnaSP v6 [84].

## 4. Conclusions

In this study, it was proven that the essential oils of *Aniba canelilla* are rich in benzenoid compounds, while *A. rosaeodora*, *A. parviflora*, and *A. terminalis* are rich in monoterpene hydrocarbons and oxygenated monoterpenes, with important variations in the relative quantity of major constituents. The primary classes of compounds showed a significant correlation with phylogenetic analysis. The *psbA-trnH* and ITS regions allowed the estimation of relatively high polymorphic sites and nucleotide diversity. The regions *rbcL* and *matK*, although very conserved, provided important information on the taxonomic levels to ordering genus and family. In general, the *psbA-trnH* and ITS genes were significant in terms of nucleotide differentiation, while the *matK* and *rcbL* genes indicated genetic similarity between the species studied. Based on the results, it was possible to verify that the genetic and chemical data are closely related in the studied *Aniba* species.

## Figures and Tables

**Table 1:** Yield and volatile composition of the *Aniba* essential oils.

Oil Yield (%)					1.5	1.0	1.6	0.9	1.8	1.6	1.3	0.7
Constituents (%)	Class	CAS Number	RI(_C_)	RI(_L_)	AC-L	AC-T	AP-L	AP-T	AR-L	AR-T	AT-L	AT-T
(3*E*)-Hexenol	O	928-97-2	848	844 ^1^	-	-	-	-	0.2	-	-	-
(3*Z*)-Hexenol	O	928-96-1	846	850 ^1^	-	-	1.0	-	-	-	-	-
1-Hexanol	O	111-27-3	860	863 ^1^	-	-	-	-	0.2	-	-	-
α-Thujene	MH	2867-05-2	828	924 ^1^	-	-	0.3	0.2	-	-	0.2	0.1
α-Pinene	MH	80-56-8	934	932 ^1^	-	2.1	10.6	4.7	-	0.3	4.3	3.8
Camphene	MH	79-92-5	945	946 ^1^	-	-	2.4	1.7	-	-	0.7	2.0
Benzaldehyde	BZ	100-52-7	954	952 ^1^	0.1	0.1	-	-	-	-	0.4	-
Sabinene	MH	3387-41-5	972	969 ^1^	-	-	-	-	-	-	-	2.2
β-Pinene	MH	18172-67-3	973	974 ^1^	-	1.3	6.4	3.5	-	0.2	1.5	3.6
Myrcene	MH	123-35-3	985	988 ^1^	-	0.4	3.2	4.1	0.3	0.4	3.6	-
α-Phellandrene	MH	99-83-2	1000	1002 ^1^	-	0.2	1.3	3.0	-	-	51.8	36.0
α-Terpinene	MH	99-86-5	1012	1014 ^1^	-	-	0.2	0.5	-	-	-	-
*cis*-*p*-menth-2-en-1-ol	OM	53399-74-9	1124	1118 ^1^	-	-	0.1	0.1	-	-	-	-
*p*-Cymene	MH	99-87-6	1020	1020 ^1^	-	0.1	1.0	0.4	-	-	12.0	7.5
Limonene	MH	5989-27-5	1024	1024 ^1^	-	-	-	-	0.2	0.3	-	-
β-Phellandrene	MH	555-10-2	1026	1025 ^1^	-	2.8	22.6	25.4	-	-	11.6	11.9
(*Z*)-β-Ocimene	MH	3338-55-4	1032	1032 ^1^	-	-	-	3.3	-	0.1	1.4	1.8
Benzene acetaldehyde	BZ	122-78-1	1037	1036 ^1^	2.7	0.5	-	-	-	-	-	-
(*E*)-β-Ocimene	MH	3779-61-1	1043	1044 ^1^	-	0.5	1.7	-	0.1	0.2	0.4	0.7
γ-Terpinene	MH	99-85-4	1056	1054 ^1^	-	-	0.6	0.7	-	-	0.2	0.1
Acetophenone	BZ	98-86-2	1061	1059 ^1^	-	-	-	-	-	-	-	-
*cis*-Linalool oxide (furanoid)	OM	23007-29-6	1071	1067 ^1^	-	-	0.3	-	5.4	2.6	0.1	-
*trans*-Linalool oxide (furanoid)	OM	41720-60-9	1089	1084 ^1^	-	-	0.4	-	4.9	2.5	-	-
Terpinolene	MH	586-62-9	1088	1086 ^1^	-	-	-	0.2	-	-	1.0	0.6
Linalool	OM	78-70-6	1102	1095 ^1^	3.9	16.1	21.9	15.2	67.9	75.3	-	19.0
*trans*-Pinene hydrate	OM	4948-29-2	1112	1119 ^1^	-	-	-	-	-	0.1	-	-
*allo*-Ocimene	MH	3016-19-1	1131	1128 ^1^	-	-	-	-	-	-	0.1	0.1
Benzene acetonitrile	BZ	140-29-4	1138	1134 ^1^	0.4	0.4	-	-	-	-	-	-
*iso*-3-Thujanol	OM	33766-31-3	1141	1134 ^1^	-	-	-	0.1	-	-	-	-
*trans*-Pinocarveol	OM	547-61-5	1141	1135 ^1^	-	-	0.2	-	-	-	-	-
Borneol	OM	6627-72-1	1168	1165 ^1^	-	-	0.5	0.4	-	-	0.1	0.5
*cis*-Linalool oxide (pyranoid)	OM	22628-11-1	1172	1170 ^1^	-	-	-	-	0.3	0.1	-	-
*trans*-Linalool oxide (pyranoid)	OM	41720-62-1	1176	1173 ^1^	-	-	-	-	0.4	0.2	-	-
Terpinen-4-ol	OM	562-74-3	1181	1174 ^1^	-	-	0.7	0.7	-	-	0.1	0.1
*p*-Cymen-8-ol	OM	1197-01-9	1188	1179 ^1^	-	-	-	-	-	-	0.1	-
Cryptone	OM	500-02-7	1190	1183 ^1^	0.1	-	0.1	-	-	-		-
α-Terpineol	OM	98-55-5	1195	1186 ^1^	0.3	0.4	3.9	3.9	0.2	0.6	0.8	0.9
*trans*-Piperitol	OM	16721-39-4	1206	1207 ^1^	-	-	-	-	-	-	-	-
Nerol	OM	106-25-2	1231	1227 ^1^	-	-	-	-	-	0.1	-	-
Geraniol	OM	106-24-1	1256	1249 ^1^	-	-	-	-	0.1	0.2	-	-
2-Phenylethyl acetate	BZ	103-45-7	1258	1254 ^1^	0.2	0.1	-	-	-	-	-	-
Thymol	OM	89-83-8	1289	1289 ^1^	-	-	-	-	-	-	-	0.1
1-Nitro-2-phenylethane	BZ	6125-24-2	1297	1294 ^1^	88.3	70.9	0.1	0.2	0.1	0.2	-	-
δ-Elemene	SH	20307-84-0	1340	1335 ^1^	-	-	0.3	0.1	-	-	-	-
α-Cubebene	SH	17699-14-8	1352	1345 ^1^	-	0.3	0.1	1.3	-	-	0.1	0.4
Eugenol	PP	97-53-0	1358	1356 ^1^	0.1	0.3	-	-	-	-	-	-
Methyl *p*-anisate	BZ	121-98-2	1374	1375 ^2^	-	-	-	-	0.2	0.1	-	-
α-Copaene	SH	3856-25-5	1379	1374 ^1^	0.1	0.3	-	0.3	1.1	0.5	0.1	0.1
β-Elemene	SH	33880-83-0	1395	1389 ^1^	-	-	0.2	0.4	0.2	0.2	0.4	0.6
7-*epi*-Sesquithujene	SH	159407-35-9	1392	1390 ^1^	-	-	-	0.3	-	-	-	0.1
(*Z*)-Caryophyllene	SH	118-65-0	1411	1408 ^1^	0.5	-	-	-	-	-	-	-
(*E*)-Caryophyllene	SH	87-44-5	1424	1417 ^1^	-	0.8	2.8	1.9	0.2	0.3	1.2	0.8
*trans*-α-Bergamotene	SH	13474-59-4	1439	1432 ^2^	-	-	-	0.1	-	-	-	-
Aromadendrene	SH	109119-91-7	1444	1439 ^1^	-	-	0.5	-	-	-	-	-
(*Z*)-β-Farnesene	SH	28973-97-9	1433	1440 ^1^	-	-	0.1	1.5	-	-	0.1	0.5
α-Himachalene	SH	3853-83-6	1447	1449 ^1^	-	-	0.2	-	-	-	-	-
(*E*)-β-Farnesene	SH	18794-84-8	1459	1454 ^1^	-	-	-	1.2	-	-		0.4
α-Humulene	SH	6753-98-6	1459	1454 ^1^	-	0.2	0.4	-	-	-	0.2	-
α-Acoradiene	SH	24048-44-0	1464	1464 ^1^	-	-	0.2	-	-	-	-	-
γ-Muurolene	SH	30021-74-0	1474	1478 ^1^	-	-	-	-	-	-	0.1	-
γ-Gurjunene	SH	22567-17-5	1480	1475 ^1^	-	-	-	-	0.3	-	-	-
γ-Curcumene	SH	28976-68-3	1483	1481 ^1^	-	-	-	0.2	-	-	-	-
Germacrene D	SH	23986-74-5	1484	1484 ^1^	-	-	0.4	0.6	-	-	0.3	0.3
β-Selinene	SH	17066-67-0	1492	1489 ^1^	-	0.3	0.1	0.6	2.9	0.5	0.2	0.1
α-Selinene	SH	473-13-2	1497	1498 ^2^	-	0.2	1.1	-	-	-	0.2	
Valencene	SH	4630-07-3	1501	1496 ^1^	-	-	-	-	2.4	0.4		0.2
Bicyclogermacrene	SH	67650-90-2	1502	1500 ^1^	-	-	1.9	0.9	-	-	-	-
α-Muurolene	SH	10208-80-7	1505	1500 ^1^	-	-	-	0.1	-	-	-	-
(*E*,*E*)-α-Farnesene	SH	502-61-4	1504	1505 ^1^	-	-	-	-	-	-	0.1	0.1
γ-Cadinene	SH	483-74-9	1512	1513 ^2^	-	-	-	-	-	-	0.1	0.1
β-Curcumene	SH	28976-67-2	1516	1514 ^1^	-	-	-	0.3	-	-	-	-
7-*epi*-α-Selinene	SH	28290-23-5	1524	1520 ^1^	-	-	0.1	0.1	-	-	-	-
δ-Cadinene	SH	483-76-1	1521	1522 ^1^	-	0.1	0.2	0.9	-	0.1	0.2	0.2
*trans*-Cadina-1,4-diene	SH	38758-02-0	1537	1533 ^1^	-	-	-	0.1	-	-	-	-
*cis*-Sesquisabinene hydrate	OS	58319-05-4	1548	1542 ^1^	-	-	-	0.2	-	-	-	0.1
Elemol	OS	639-99-6	1554	1548 ^1^	-	-	0.2	2.0	-	-	0.9	1.3
Germacrene B	SH	15423-57-1	1559	1559 ^1^	-	-	-	-	0.1	-	-	-
(*E*)-Nerolidol	OS	40716-66-3	1567	1561 ^1^	-	-	0.2	0.6	0.1	0.2	0.1	0.3
Palustrol	OS	5986-49-2	1575	1567 ^1^	-	-	0.2	-	-	-	-	-
Spathulenol	OS	6750-60-3	1584	1577 ^1^	-	0.1	3.4	0.9	1.0	0.6	0.1	0.2
Caryophyllene oxide	OS	1139-30-6	1587	1582 ^1^	2.4	0.1	1.7	0.7	1.6	1.0	0.2	0.3
Viridiflorol	OS	552-02-3	1598	1592 ^1^	-	-	-	0.1	-	-	-	-
Cubeban-11-ol	OS	864875-70-7	1600	1595 ^1^	-	-	0.3	-	-	-	-	-
Guaiol	OS	489-86-1	1603	1600 ^1^	-	-	-	1.0	-	-	0.5	0.4
Rosifoliol	OS	63891-61-2	1606	1600 ^1^	-	-	0.4	0.4	-	-	-	0.1
Khusimone	OS	30557-76-7	1603	1604 ^1^	-	-	0.3	-	-	-	-	-
β-Atlantol	OS	38142-56-2	1609	1608 ^1^	-	-	0.2	-	-	-	-	-
Humulene epoxide II	OS	19888-34-7	1616	1608 ^1^	-	-	-	-	0.4	0.1	0.1	-
Junenol	OS	472-07-1	1619	1618 ^1^	-	-	-	-	0.2	0.3	-	-
10-*epi*-γ-Eudesmol	OS	15051-81-7	1626	1622 ^1^	-	-	-	0.2	-	-	-	-
*epi*-γ-Eudesmol	OS	117066-77-0	1627	1624 ^2^	-	-	-	0.3	-	-	-	-
Eremoligenol	OS	10219-71-3	1629	1629 ^1^	-	-	0.3	-	-	-	-	0.2
γ-Eudesmol	OS	1209-71-8	1638	1630 ^1^	-	-	1.6	4.5	-	-	-	-
β-Acorenol	OS	28400-11-5	1644	1636 ^1^	-	-	0.6	-	-	-	-	-
*allo*-Aromadendrene epoxide	OS	85760-81-2	1644	1639 ^1^	0.3	-	-	0.3	-	-	-	-
Caryophylla-4 (12),8(13)-dien-5β-ol	OS	19431-80-2	1642	1639 ^1^	-	-	-	-	0.1	0.1	-	-
*epi*-α-Muurolol	OS	19912-62-0	1646	1640 ^1^	-	-	-	1.0	-	-	-	-
α-Muurolol	OS	19435-97-3	1651	1644 ^1^	-	-	-	-	-	-	0.1	-
Cubenol	OS	21284-22-0	1651	1645 ^1^	-	-	-	-	-	0.3	-	-
Khusilal	OS	2221-68-3	1651	1647 ^1^	-	-	0.1	-	0.1	-	-	-
β-Eudesmol	OS	473-15-4	1656	1649 ^1^	-	-	0.4	1.7	-	-	0.8	0.4
α-Eudesmol	OS	473-16-5	1659	1652 ^1^	-	-	0.6	2.3	-	-	1.0	-
Pogostol	OS	21698-41-9	1661	1651 ^1^		0.7	-	-	-	-	-	-
*neo*-Intermedeol	OS	5945-72-2	1660	1658 ^1^	0.1	-	-	-	-	0.6	-	-
Selin-11-en-4α-ol	OS	16641-47-7	1660	1658 ^1^	-	-	-	-	0.9	-	-	-
14-Hydroxy-(*Z*)-caryophyllene	OS	78683-81-5	1663	1666 ^1^	-	-	-	-	-	0.2	-	-
14-Hydroxy-9-*epi*- (*E*)-caryophyllene	OS	79768-25-5	1667	1668 ^1^	0.2	-	0.3		0.2	0.9	-	-
Bulnesol	OS	22451-73-6	1672	1670 ^1^	-	-	0.2	1.3	-	-	0.4	0.4
*epi*-β-Bisabolol	OS	235421-59-7	1675	1670 ^1^	-	-	-	0.8	-	-	-	0.1
(*Z*)-α-Santalol	OS	115-71-9	1677	1674 ^1^	-	-	-	-	0.4	1.4	-	-
Khusinol	OS	24268-34-6	1672	1679 ^1^	-	-	-	0.1	0.2	-	-	-
α-Bisabolol	OS	515-69-5	1687	1685 ^1^	-	-	-	0.8	-	-	-	0.1
14-Hydroxy-α-humulene	OS	75678-90-9	1713	1713 ^1^	-	-	-	-	1.2	0.1	-	-
*iso*-Longifolol	OS	1139-17-9	1728	1728 ^1^	-	-	-	-	2.6	3.1	-	-
(*Z*)-Lanceol	OS	10067-28-4	1752	1760 ^1^	-	-	-	-	0.2	-	-	-
β-Acoradienol	OS	149496-35-5	1762	1762 ^1^	-	-	-	-	0.1	0.3	-	-
Benzyl benzoate	BZ	120-51-4	1769	1772 ^2^	-	-	-	0.1	0.3	0.9	-	0.1
Monoterpene Hydrocarbons					-	7.4	50.3	47.7	0.6	1.5	88.8	70.4
Oxygenated monoterpenoids					4.3	16.5	28.1	20.4	79.2	81.7	1.2	20.6
Sesquiterpene Hydrocarbons					0.6	2.2	8.6	10.9	7.2	2.0	3.3	3.9
Oxygenated sesquiterpenoids					3.0	0.9	11.0	19.2	9.3	9.2	4.2	3.9
Benzenoid compounds					91.8	72.3	0.1	0.3	0.4	1.2	0.4	0.1
Others					-	-	1.0	-	0.4	-	-	-
Total					99.7	99.3	99.1	98.5	97.3	95.6	97.9	98.9

RI_(C)_ = calculated retention index; RI_(L)_ = literature retention index; ^1^ [41]; ^2^ [42]; AC: *A. canelilla*; AP: *A. parviflora*, AR: *A. rosaeodora*, AT: *A. terminalis*, L: Leaves, T: Twigs, MH: Monoterpene hydrocarbons, OM: Oxygenated monoterpene, SH: Sesquiterpene hydrocarbons, OS: Oxygenated sesquiterpenoids, BZ: Benzenoids. Bold = main constituents above 5%.

**Table 2:** Molecular characteristics of the four markers evaluated for *Aniba* species.

DNA Markers	Alignment Length (bp)	Number of Polymorphic Sites	Total Number of Sites *
*matK*	286	-	300
*rbcL*	833	5	829
*psbA-trnH*	471	27	401
ITS	249	26	239
*matK* + *rbcL* + *psbA* − *trnH*	1590	32	1508
*matK* + *rbcL* + ITS	1368	31	1346
*matK* + *rbcL* + *psbA* − *trnH* + ITS	1839	58	1747

* Excluding sites with gaps/missing data.

**Table 3:** Data from *Aniba* species.

Species	Vouchers	Plant Material	Sample Codes
*Aniba canelilla* (Kunth) Mez.	MG135105	Leaves	AC-L
		Twigs	AC-T
*Aniba parviflora* (Meisn.) Mez.	MG227333	Leaves	AP-L
		Twigs	AP-T
*Aniba rosaeodora* Ducke	MG229347	Leaves	AR-L
		Twigs	AR-T
*Aniba terminalis* Ducke	MG172694	Leaves	AT-L
		Twigs	AT-T

**Table 4:** Primer sequences applied in DNA amplification of *Aniba* species and its experimental conditions.

Region	Primers	Sequence(5′–3′)	Amplification Protocol
ITS	ITS 1F	GAGCTCCGAACAAACCCTCT	95 °C 7 min; 95 °C 1 min, 52 °C 1 min, 72 °C 1 min, 35 cycles; 72 °C 7 min
	ITS 1R	AAGACTCGATGGTTCACGGG	
	ITS 2F	CCCGTGAACCATCGAGTCTTT	
	ITS 2R	GACGGCTCGCCTCTCAAC	
*matK* ^a^	*matK-Lau001*	TCCTTTCTTGAGCGAACACA	5 °C 7 min; 95 °C 1 min, 56 °C 1 min, 72 °C 1 min, 35 cycles; 72 °C 7 min
	*matK-Lau002*	CTGACAAATCGGACCGAAAC	
*psbA* ^b^	*psbA3*_f F	GTTATGCATGAACGTAATGCT	5 °C 7 min; 95 °C 1 min, 56 °C 1 min, 72 °C 1 min, 35 cycles; 72 °C 7 min
*trnH* ^c^	*trnHf*_05 R	CGCGCATGGTGGATTCACAATCC	
*rbcL*	*rbcL* 1F	GGACAACTGTGTGGACCGAT	95 °C 7 min; 95 °C 1 min, 61 °C 1 min, 72 °C 1 min, 35 cycles; 72 °C 7 min
	*rbcL* 1R	AAACGGTCTCTCCAACGCAT	
*rbcL*	*rbcL* 2F	ATGCGTTGGAGAGACCGTTT	95 °C 5 min; 95 °C 1 min, 53 °C 1 min, 72 °C 1 min, 30 cycles; 72 °C 7 min
	*rbcL* 2R	AAAGTGATGTCCCGTTCCCC	
*ycf*1 ^d^	*ycf*1bF	TCTCGACGAAAATCAGATTGTTGTGAAT	95 °C 5 min; 95 °C 1 min, [50–57 °C] 1 min, 72 °C 1 min, 30 cycles; 72 °C 7 min
	*ycf*1bR	ATACATGTCAAAGTGATGGAAAA	

^a^ [71]; ^b^ [76], ^c^ [75], ^d^ [48].

**Table 5:** GenBank accession numbers of *Aniba* species collected in the Amazon.

Species	ITS	*psbA-trnH*	*rbcL*	*matK*
*Aniba canelilla* (Kunth) Mez.	MW489499	MW512551	MW512547	MW512555
*Aniba parviflora* (Meisn.) Mez.	MW489500	MW512552	MW512548	MW512556
*Aniba rosaeodora* Ducke	MW489501	MW512553	MW512549	MW512557
*Aniba terminalis* Ducke	MW489502	MW512554	MW512550	MW512558

## Data Availability

Publicly available gene sequence datasets were analyzed in this study. These data can be found here: https://www.ncbi.nlm.nih.gov/genbank/, accessed on 20 December 2020; accession numbers: *Aniba canelilla* (MW489499, MW512551, MW512547, and MW512555), *A. parviflora* (MW489500, MW512552, MW512548, and MW512556), *A. rosaeodora* (MW489501, MW512553, MW512549, and MW512557), and *A. terminalis* (MW489502, MW512554, MW512550, and MW512558).

## Supplementary Materials

The following are available online, Figure S1. Maximum likelihood tree based on *rbcL* sequences of species of *Aniba* and *Laurus nobilis* (outgroup). Bootstrap support values (67%) are shown above the branches. Figure S2. Bayesian inference tree based on *rbcL* sequences of species of *Aniba* and *Laurus nobilis* (outgroup). Bootstrap support values (≥0.7) are shown above the branches. Figure S3. Maximum likelihood tree based on *matK* sequences of species of *Aniba* and *Laurus nobilis* (outgroup). Bootstrap support values (34%) are shown above the branches. Figure S4. Bayesian inference tree based on *matK* sequences of species of *Aniba* and *Laurus nobilis* (outgroup). Figure S5. Maximum likelihood tree based on ITS sequences of species of *Aniba* and *Laurus nobilis* (outgroup). Bootstrap support values (≥33%) are shown above the branches. Figure S6. Bayesian inference tree based on ITS sequences of species of *Aniba* and *Laurus nobilis* (outgroup). Bayesian posterior probabilities (1) are shown above the branches. Figure S7. Maximum likelihood tree based on *psbA-trnH* sequences of species of *Aniba* and *Laurus nobilis* (outgroup). Bootstrap support values (≥33%) are shown above the branches. Figure S8. Bayesian inference tree based on *psbA-trnH* sequences of species of *Aniba* and *Laurus nobilis* (outgroup). Bayesian posterior probabilities (1) are shown above the branches.

## Sample Availability

Samples of this work are available from the authors.
