# Supplementary material for: Essential Oil Composition and DNA Barcode and Identification of Aniba species (Lauraceae) Growing in the Amazon Region
Source: Molecules. 2021 Mar 29;26(7):1914. doi: 10.3390/molecules26071914 (PMC8036375; doi:10.3390/molecules26071914)
Supplement: Supplementary file 1 [file molecules-26-01914-s001.pdf]

Supplementary Materials

Essential Oil Composition and DNA Barcode and Identification of *Aniba* species (Lauraceae) Growing in the Amazon Region

Júlia Karla A. M. Xavier [1], Leonardo Maia [1], Pablo Luis B. Figueiredo [2], Adriana Folador [3], Alessandra R. Ramos [4], Eloisa H. Andrade [5], José Guilherme S. Maia [6], William N. Setzer [7,8] and Joyce Kelly R. da Silva [1,8*]

[1] Programa de Pós-Graduação em Química, Instituto de Ciências Exatas e Naturais, Universidade Federal do Pará, 66075-900 Belém, Brazil; julia.xavier@icen.ufpa.br (J.K.X.); leo_henriquemaia@hotmail.com (L.H.M.); joycekellys@ufpa.br (J.K.R.S.)
[2] Departamento de Ciências Naturais, Centro de Ciências Sociais e Educação, Universidade do Estado do Pará, Belém, PA 66050-540, Brazil. pablo.figueiredo@uepa.br (P.L.F.)
[3] Laboratório de Genômica e Bioinformática, Centro De Genômica e Biologia de Sistemas, Universidade Federal do Pará, 66075-900, Belém, Brazil. adrianarc@ufpa.br (A.F.).
[4] Instituto de Estudos em Saúde e Biológicas, Universidade Federal do Sul e Sudeste do Pará, Marabá, PA, Brazil; rezende@unifesspa.edu.br (A.R).
[5] Coordenação de Botânica, Museu Paraense Emílio Goeldi, Belém, PA, Brazil; eloisa@museu-goeldi.br (E.A.).
[6] Programa de Pós-Graduação em Química, Universidade Federal do Maranhão, São Luís, MA, Brazil; gmaia@ufpa.br (J.G.M.).
[7] Department of Chemistry, University of Alabama in Huntsville, Huntsville, AL 35899, USA; wsetzer@chemistry.uah.edu (W.N.S.)
[8] Aromatic Plant Research Center, 230 N 1200 E, Suite 102, Lehi, UT 84043, USA
* Correspondence: joycekellys@ufpa.br (J.K.R.S.); Programa de Pós-Graduação em Química, Universidade Federal do Pará, 66075-900 Belém, PA, Brazil.

Maximum Likelihood and Bayesian Inference tree based on *rbcL* sequences.

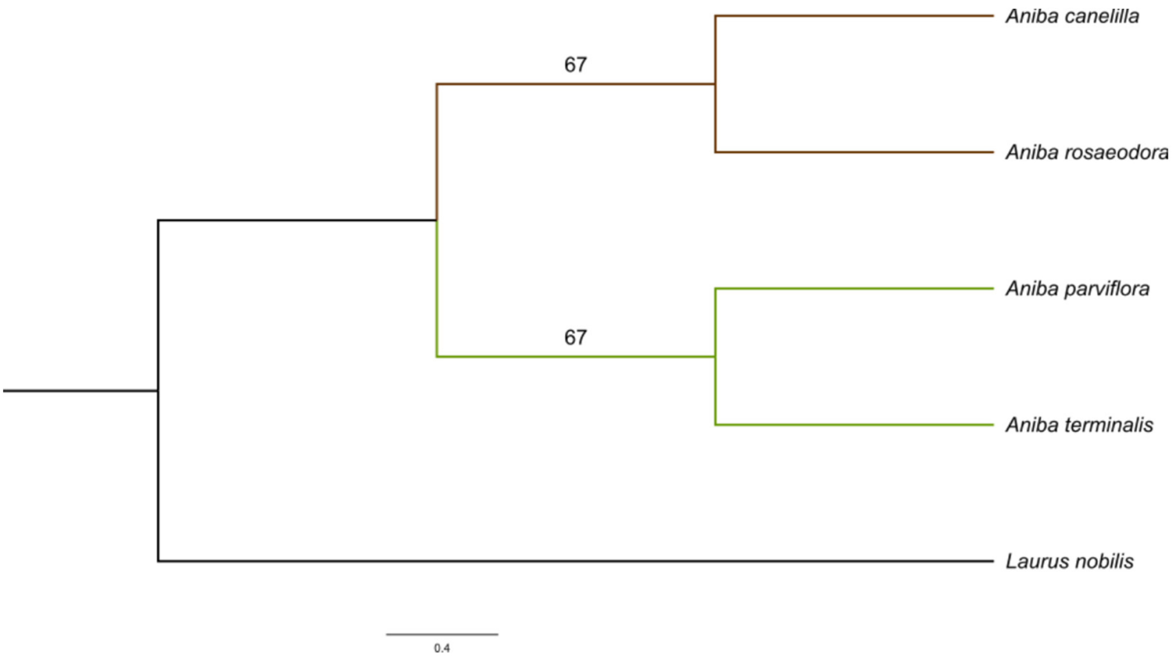

Figure S1. Maximum Likelihood tree based on *rbcL* sequences of species of *Aniba* and *Laurus nobilis* (outgroup). Bootstrap support values (67%) are shown above the branches.

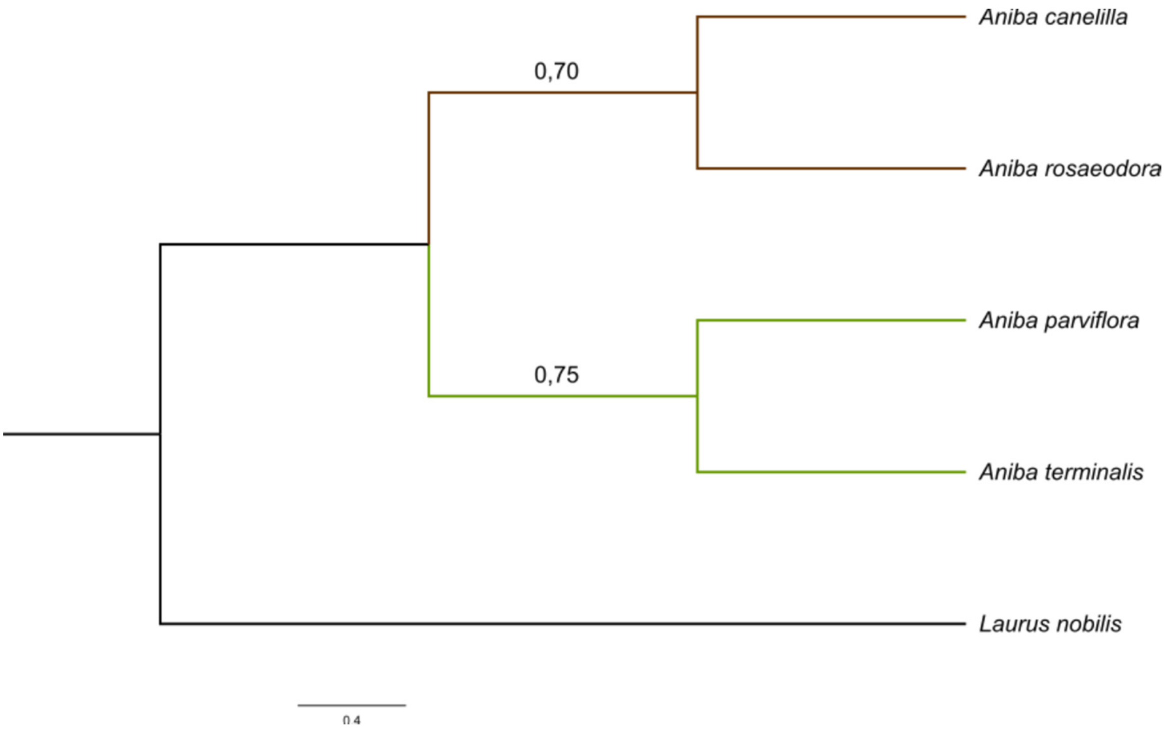

Figure S2. Bayesian Inference tree based on *rbcL* sequences of species of *Aniba* and *Laurus nobilis* (outgroup). Bootstrap support values (≥0,7) are shown above the branches.

Maximum Likelihood and Bayesian Inference tree based on *matK* sequences.

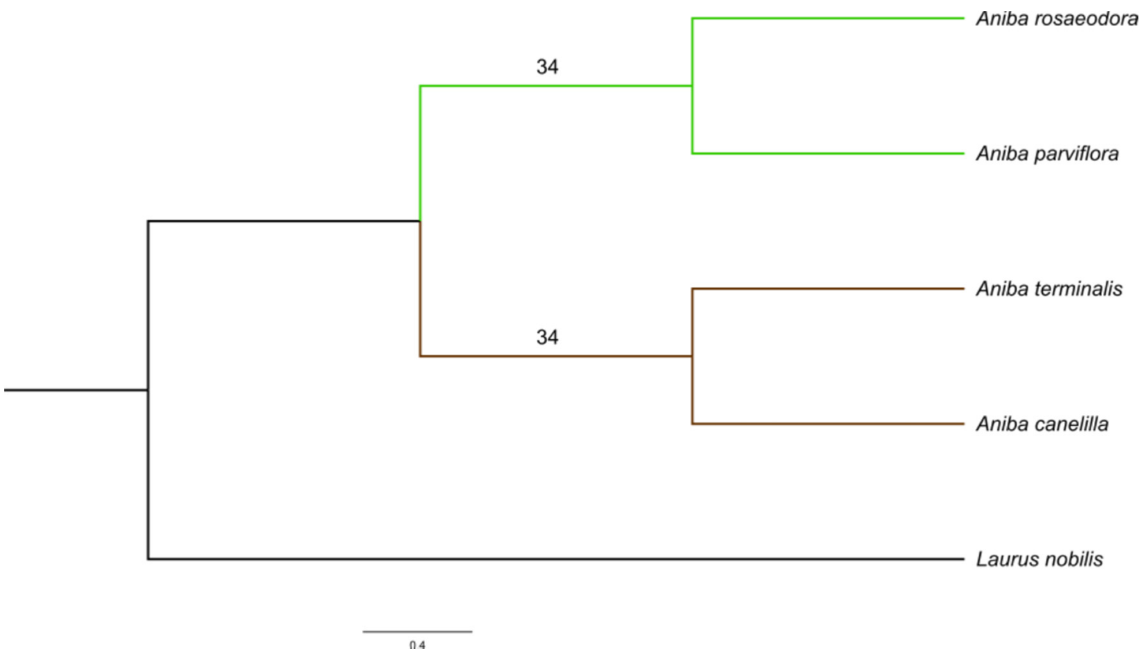

Figure S3. Maximum Likelihood tree based on *matK* sequences of species of *Aniba* and *Laurus nobilis* (outgroup). Bootstrap support values (34%) are shown above the branches.

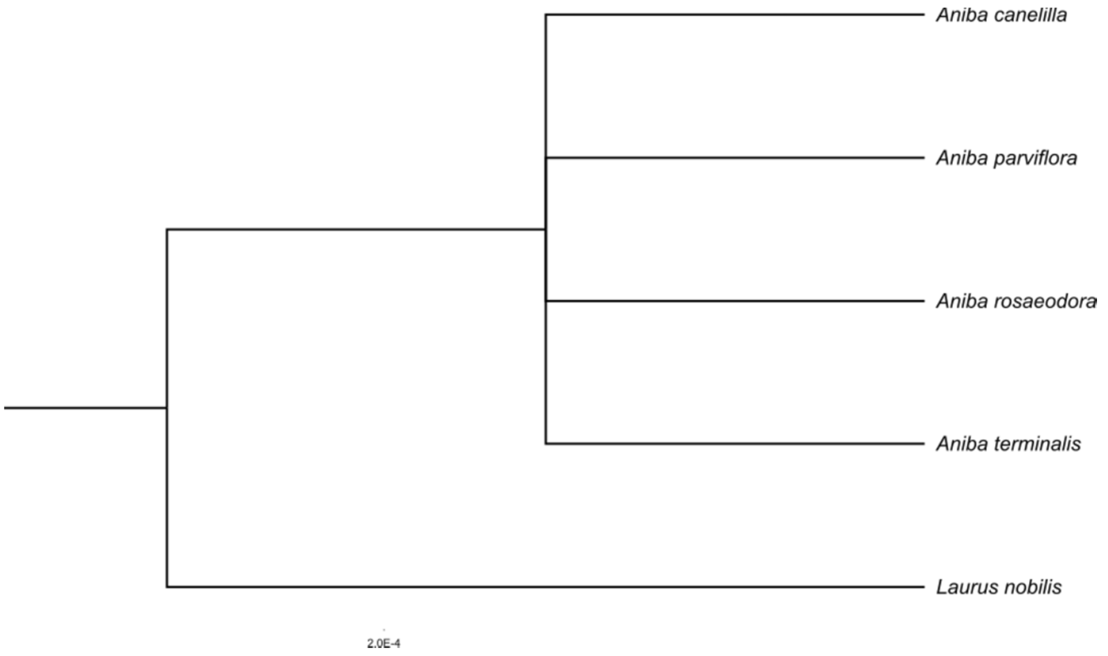

Figure S4. Bayesian Inference tree based on *matK* sequences of species of *Aniba* and *Laurus nobilis* (outgroup).

Maximum Likelihood and Bayesian Inference tree based on ITS sequences.

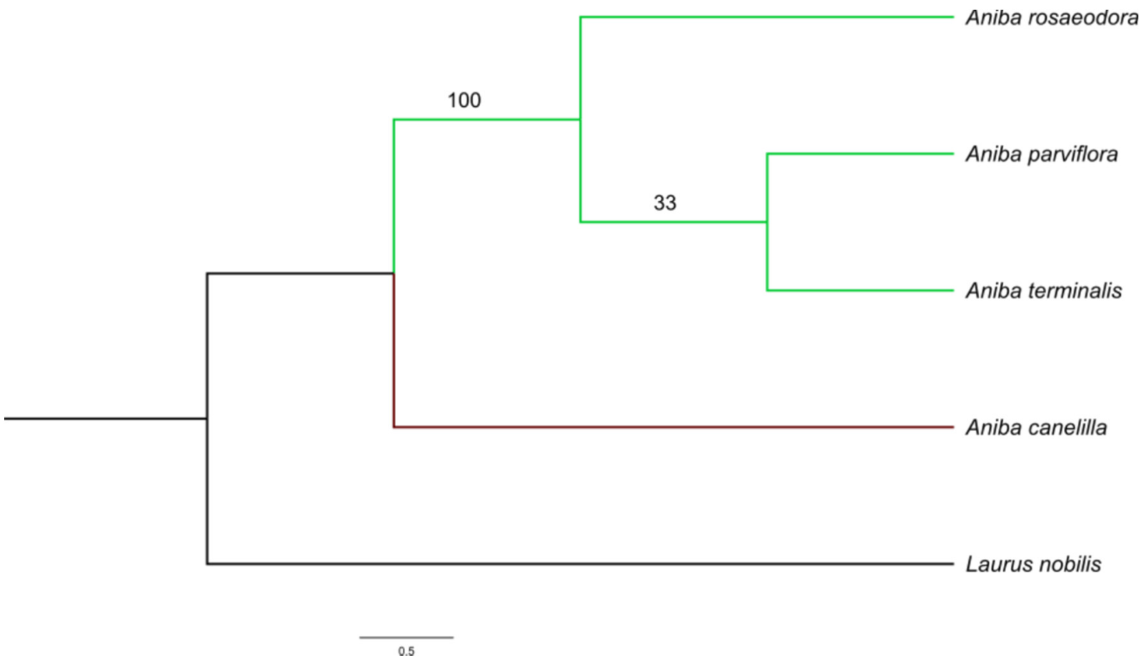

Figure S5. Maximum Likelihood tree based on ITS sequences of species of *Aniba* and *Laurus nobilis* (outgroup). Bootstrap support values (≥33%) are shown above the branches.

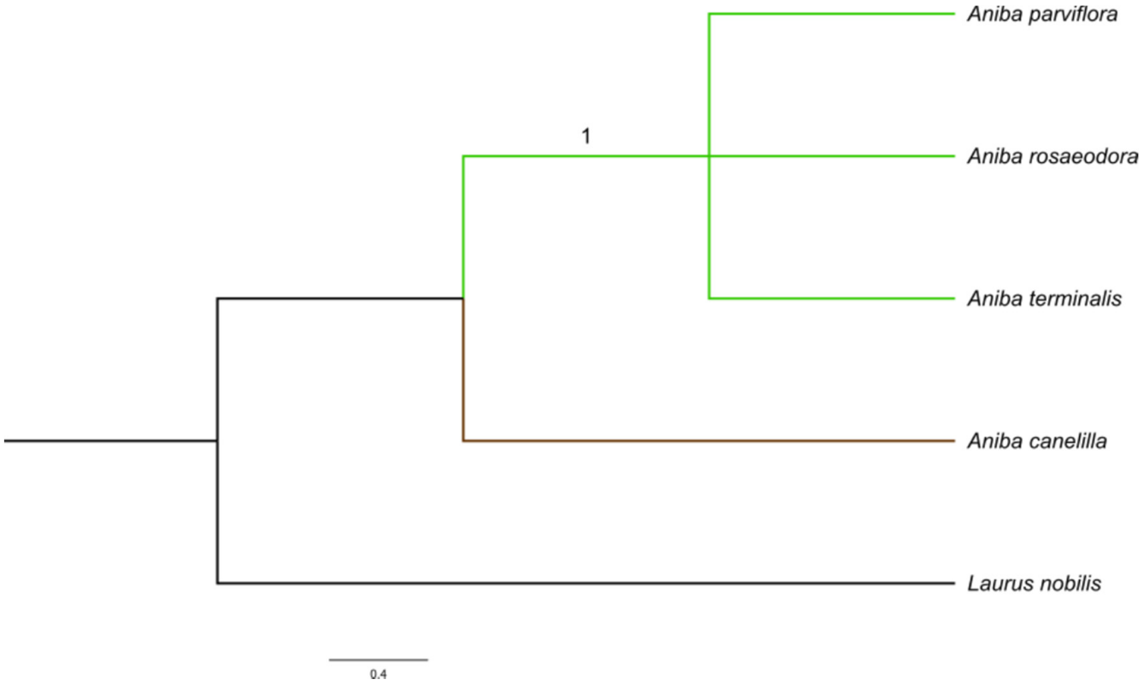

Figure S6. Bayesian Inference tree based on ITS sequences of species of *Aniba* and *Laurus nobilis* (outgroup). Bayesian posterior probabilities (1) are shown above the branches.

Maximum Likelihood and Bayesian Inference tree based on *psbA-trnH* sequences

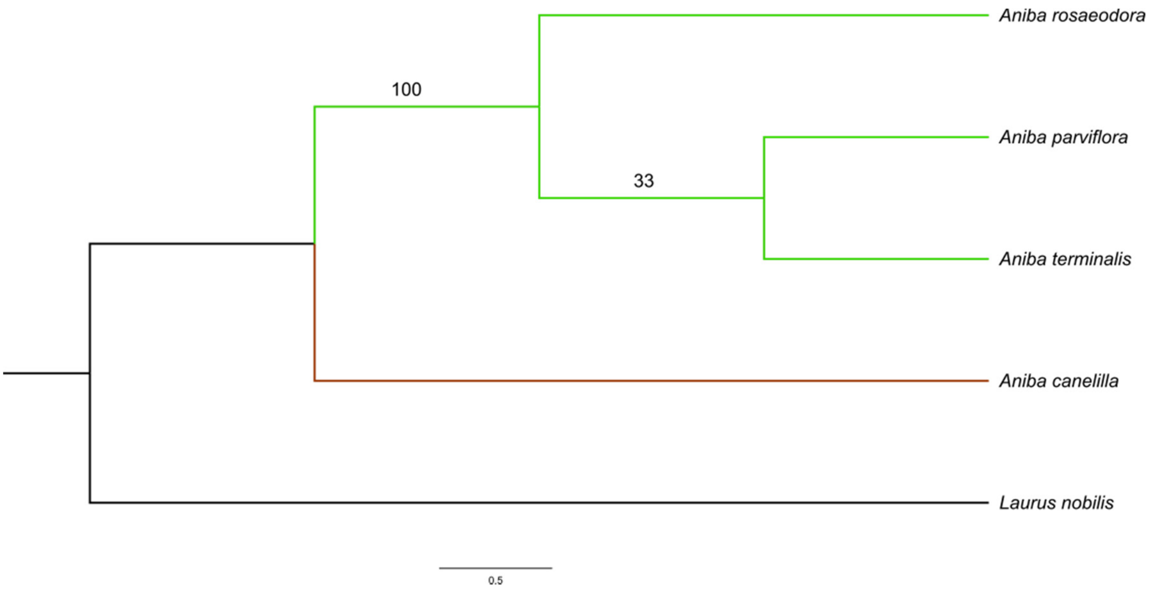

Figure S7. Maximum Likelihood tree based on *psbA-trnH* sequences of species of *Aniba* and *Laurus nobilis* (outgroup). Bootstrap support values (≥33%) are shown above the branches.

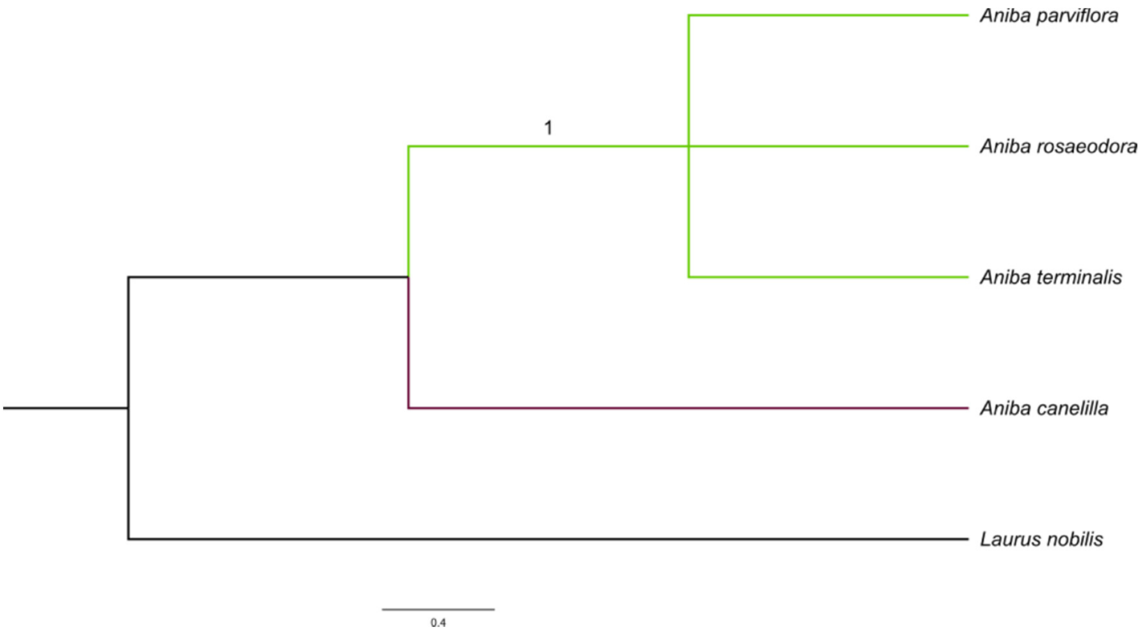

Figure S8. Bayesian Inference tree based on *psbA-trnH* sequences of species of *Aniba* and *Laurus nobilis* (outgroup). Bayesian posterior probabilities (1) are shown above the branches.
